# Repeatability of Knee Extension Muscle Endurance Between 20% and 40% of One Repetition Maximum

**DOI:** 10.3390/muscles5020026

**Published:** 2026-04-13

**Authors:** Sam J. Hillen, Matthew D. Fliss, Cameron J. Mitchell

**Affiliations:** Ageing, Nutrition, Exercise, and Muscle Metabolism Laboratory, School of Kinesiology, Faculty of Education, The University of British Columbia, Vancouver, BC V6T 1Z4, Canada; sjhillen@student.ubc.ca (S.J.H.); matthew.fliss@ubc.ca (M.D.F.)

**Keywords:** fatigue curve, low load, intraclass correlation coefficient, standard error of the measurement, smallest detectable difference

## Abstract

Dynamic muscular endurance, the ability to lift a submaximal load until task failure, is a common measure in both cross-sectional and training studies. However, the repeatability of low-load muscular endurance in the knee extensors has not been well established. Establishing reliability metrics is essential to ensure that observed differences reflect true physiological changes rather than measurement error. The purpose of this study was to quantify the repeatability of low-load dynamic knee extensions performed to task failure. Forty healthy adults completed three visits, each consisting of one set of knee extensions at 20%, 30%, and 40% of one repetition maximum (1RM) to assess relative muscular endurance, and three sets at 20% 1RM on the contralateral leg to assess the impact of fatigue within a single session (fatigue curve). Intraclass correlation coefficients (ICCs), standard error of the measurement, and smallest detectable difference (SDD) were calculated. Repeatability ranged from moderate to excellent across conditions (ICC = 0.77–0.94). Lower loads and later sets demonstrated reduced repeatability compared with heavier loads and earlier sets. These results indicate that researchers and practitioners should consider load and fatigue curve effects in protocol design and SDDs when interpreting the meaningfulness of individual changes in knee extension muscular endurance.

## 1. Introduction

Muscular endurance can be defined as one’s ability to resist muscular fatigue when performing submaximal dynamic muscular contractions [1]. Muscular endurance can be expressed as the number of repetitions that can be performed with a given load, either as a relative percentage of an individual’s one repetition maximum (1RM) or absolutely at a fixed load [1]. The relationship between relative load and the number of repetitions that can be completed (REPS~%1RM relationship) is a negatively sloped, non-linear monotonic function that is often modelled using hyperbolic, cubic or exponential equations [2,3,4], where heavier loads yield earlier task failure. For the knee-extension exercise, higher relative load muscular endurance is positively correlated with 1RM strength due to contraction-induced blood flow occlusion, whereas at lighter relative loads (≲40% 1RM), performance becomes more reliant on aerobic metabolism (e.g., oxygen delivery and mitochondrial content) [5]. Muscular endurance also decreases following repeated sets of dynamic resistance exercise, plateauing in the fifth set at approximately 45% of initial muscular endurance; this relationship has been termed the ‘fatigue curve’ [6].

Observed performance in exercise tasks vary day to day due to a combination of technical error and physiological variability, possibly leading to over-interpretation of a random effect or standard measurement error [7]. It is therefore imperative that researchers understand and report the magnitude of this variability to ensure that observed differences are meaningful rather than random [8]. Common measures of repeatability in sport science include the standard error of the measurement (SEM), smallest detectable difference (SDD), and the intraclass correlation coefficient (ICC). The SEM quantifies the typical observed error within an individual and can be conceptualized as the noise within an individual’s score [7]. The SDD instead quantifies the smallest real measurement difference that can be detected with a measure and can be conceptualized as the smallest individual score change that demonstrates a real effect [9]. Lastly, the ICC estimates the test–retest reliability coefficient of a particular measure, providing a relative repeatability metric that can be compared across conditions [10]. These measures of repeatability allow for research to remain repeatable, with findings being better understood and contextualized.

As relative load increases, muscle force of contraction must increase to meet the task’s demands. Muscle modelling has correlated this increase in force with a rise in intramuscular pressure (IMP) [11,12], likely responsible for the inverse relationship between relative load and muscle oxygenation during resistance exercise [13]. When muscle blood flow is externally occluded with a pressurized cuff simulating the effects of elevated IMP, muscle oxygenation decreases and fibre-type specific metabolism is altered [14,15] in a dose-specific pattern [16]. Indeed, dynamic knee extension muscular endurance decreases when leg blood flow is occluded despite similar levels of effort [17,18]. However, this effect only exists at lower relative loads (<50% 1RM); thus, the load at which blood flow occlusion no longer reduces performance is known as critical occlusion tension (COT). Muscle radial expansion in response to loads above the COT is sufficient to occlude blood flow and severely limit the potential for aerobic metabolism [17]. Recently, one study demonstrated a COT at approximately 40% 1RM where muscular endurance becomes influenced by aerobic training status [5]. Taken together, these data suggest that when the relative load is light enough (below the COT), capillary blood flow and oxygen supply are sufficient to meet the task’s demands.

Although both interindividual differences in the REPS~%1RM relationship [2] and repeatability of 1RM tests [19] have been thoroughly investigated, little work has reported the repeatability of muscular endurance performance of the knee extensors. Muscular endurance can be tested at different loads—usually less than 60% 1RM [1]—and multiple tests are often performed on the same day to model the REPS~%1RM relationship. However, it is currently unclear how differences in relative load and the addition of multiple trials might alter the repeatability of dynamic knee extension muscular endurance. Furthermore, to the best knowledge of the authors, no past work has shown whether muscular endurance performance below COT is repeatable. Therefore, the present study sought to determine the repeatability of low-load dynamic resistance exercise performed to volitional failure. The primary aim was to calculate the SDD of muscular endurance at 20%, 30%, and 40% 1RM and of the absolute muscular endurance fatigue curve at 20% 1RM. Secondary aims included measuring the lower-load ICC, SEM, REPS~%1RM relationship, and 20% 1RM muscular endurance fatigue curve. Exploratory analyses into the effects of potential confounding variables including sex and training status were also conducted.

## 2. Materials and Methods

### 2.1. Experimental Approach to the Problem

To investigate the repeatability of low-load muscular endurance, a repeated-measures, quasi-experimental, within- and between-subject design was used. All exercises were performed on a unilateral knee extension exercise machine (C-605 Unilateral Leg Extension, Atlantis Strength, Laval, QC, Canada). Following a 1RM strength testing visit, muscular endurance was determined at 20%, 30%, and 40% 1RM performed on the right leg in a random order. The fatigue curve was determined with three sets (S1, S2, and S3) at 20% 1RM performed on the left leg. Although leg dominance was not considered, the allocation was kept consistent across all visits, simplifying the study protocol while preserving within-subject consistency. The following potential confounding variables were recorded or standardized: sex and training status were included in the model as a factor and covariate, respectively, previous sessions’ diet and sleep logs were provided to participants and they were asked to approximately mimic them following each visit, and contraction tempo and inter-set rest periods were controlled, as described in Section 2.3.

### 2.2. Participants

Healthy young males (n = 20) and females (n = 20) between the ages of 19 to 30 voluntarily participated in this study. Descriptive statistics and visit spacing for the participants are shown in Table 1. Participants were included and classified as healthy based on the exclusion criteria of (1) any major uncontrolled cardiovascular, muscular, metabolic, and/or neurological disorders, (2) any medical condition impacting the ability to participate in maximal exercise, (3) type one or type two diabetes, (4) diagnosis of cancer or undergoing cancer treatment in the past 12 months, (5) pharmacological therapy with any drugs known to alter skeletal muscle metabolism, or (6) current use of cigarettes or other nicotine devices. All participants were recruited in the University of British Columbia (UBC) Vancouver area via convenience sampling. The study was approved by the UBC Research Ethics Board (H23-03127) and was conducted following the Declaration of Helsinki.

### 2.3. Procedures

There was a total of four visits per participant. The first visit served to measure the participant’s 1RM, record their average moderate–vigorous physical activity (MVPA), low-intensity physical activity (LIPA), and resistance training (RT) minutes per week in the past year, familiarize them with the exercise, and randomize their loading order. Signed consent was obtained for all participants after screening for participation using a physical activity readiness questionnaire [20]. The 1RM strength test was performed in the same manner as Fliss et al. [5]. One familiarization set of 8–12 repetitions at both 20% and 40% 1RM was performed in that order, with the range of motion and contraction tempo described below. Loading order was randomized by participants selecting a pre-made folded slip of paper with their respective allocation from an opaque container, and was maintained for all subsequent visits, while counterbalancing for sex. This was accomplished by producing four copies of each of the six possible loading orders (20/30/40, 20/40/30, 30/20/40, 30/40/20, 40/20/30, and 40/30/20), selecting one for each participant’s ID, and matching across sexes; two loading orders were left with an additional male and female allocation. Due to the design of the study, neither the researcher nor the participants could be blinded to the exercise order or current exercise condition.

The following three visits required participants to complete a single set at loads of 20%, 30% and 40% 1RM on their right leg (RME), and three sets at 20% 1RM on their left leg (AME_FC20%_) of knee extension exercise to volitional failure. Volitional failure was defined as the participant (1) choosing to stop, (2) the inability to maintain tempo for two consecutive repetitions, or (3) not completing the full range of motion for two consecutive repetitions. Consistent verbal encouragement was provided by the same researcher (SH) across all testing visits, loads, and sets, using language such as ‘keep fighting,’ ‘still strong,’ and ‘push, push, push.’ Visits were separated by at least 48 h, and participants refrained from strenuous exercise 24 h and alcohol 12 h before each visit. After arriving at the lab, participants filled out a 24 h sleep and diet log, estimating the quality and number of hours slept the previous night. This form’s purpose was to encourage participants to eat and sleep similarly for each testing visit in addition to the verbal instruction given by the researchers. Participants then completed 2 sets of 8–12 repetitions at 40% 1RM with any ‘controlled’ tempo as a warm-up, followed by 3 min of rest. All 6 sets were then performed, alternating between the RME leg and the AME_FC20%_ leg, with 10 min of rest between sets to minimize cross-over fatigue. This allowed for 20 min of rest between sets on the same leg, minimizing within-leg fatigue while preserving study feasibility. Repetitions were counted if the knee angle reached within 10 degrees of full extension at the top of the repetition, the shank was perpendicular to the ground at the bottom of the repetition, and the tempo was maintained (2 s concentric and 2 s eccentric).

### 2.4. Statistical Analyses

Statistical analysis was performed using SPSS version 29.0.1.0 (IBM Corp., New York, NY, USA, 2023). Muscular endurance differences in the number of repetitions performed at different loads were analyzed with a four-way mixed model (load × order × visit × sex), while AME_FC20%_ differences were analyzed with a three-way mixed model (set × visit × sex). All factors were repeated except for sex. Repeatability was analyzed with a two-way mixed-effects, absolute-agreement, single-score ICC, reported alongside its 95% confidence interval (CI), SEM, and SDD. ICCs were assessed with the following criteria listed in Koo and Li [10]: poor < 0.50, moderate 0.50–0.75, good 0.75–0.90, and excellent > 0.90. SEM was calculated using Equation (1), as described by Hopkins [7]:

(1) $$SEM=SD\sqrt{1-ICC},$$

where SD is the average of the standard deviations of all participants in each condition and ICC is the intraclass correlation coefficient of each condition. SDD was calculated using Equation (2), as described by Beckerman et al. [9]:

(2) SDD=1.96×√2×SEM,

where SEM is the standard error of the measurement of each condition. Two separate mixed-model analyses were also run on the RME (load × sex) and AME_FC20%_ (set × sex) coefficients of variation (CVs) in number of repetitions performed to test for differences in repeatability. MVPA and RT were set as covariates and Pearson’s correlation coefficients were calculated to examine their associations with RME and AME_FC20%_. Significance was set at *p* ≤ 0.05.

## 3. Results

Males were stronger than females on both the right (M: 51.4 ± 10.3 kg; F: 37.2 ± 6.8 kg) and left (M: 51.9 ± 10.2 kg; F: 36.6 ± 6.5 kg) legs (*p* < 0.001). Neither RME (*p* = 0.217) or AME_FC20%_ (*p* = 0.711) changed across the three visits. Simpler models are generally preferable to more complex models, improve interpretability, and can reduce the likelihood of type II error [21]. Visit was thus removed as a fixed factor and included as a covariate for all subsequent analyses to increase model parsimony.

### 3.1. Repeatability of Muscular Endurance

**Figure 1:**
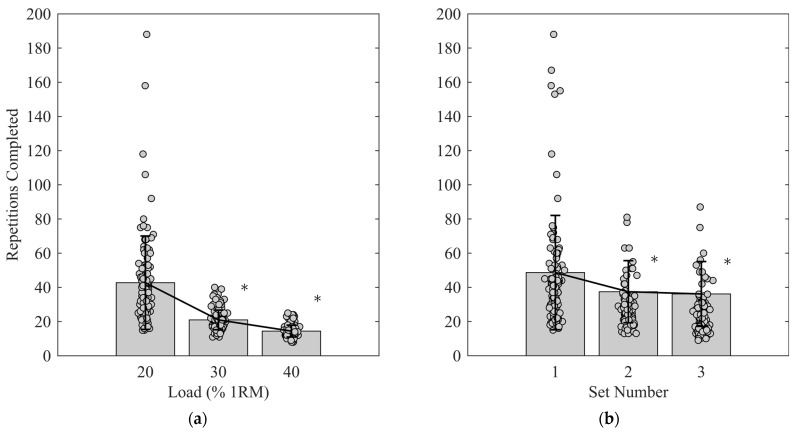

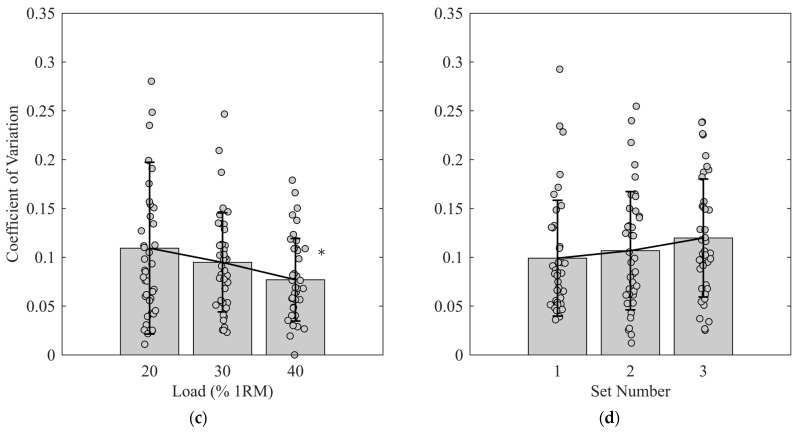
Muscle endurance and coefficient of variation (CV) across loads and sets: (**a**) number of repetitions performed at each of the three external relative loads. * Significant difference from next lightest load; (**b**) number of repetitions performed at each fatigue curve set (AME_FC20%_). * Significant difference from set 1; (**c**) coefficients of variation (CV) in each of the three external relative loads. * Significant difference from 20% 1 repetition maximum (1RM); (**d**) CVs in each set of AME_FC20%_.

All loads and sets ranged from good to excellent repeatability, as described by Koo and Li [10] (Table 2). SEM and SDD are reported in Table 2. Repeatability decreased with lighter loads and later sets, as indicated by the reliability metrics (Table 2). The coefficient of variation (CV) in number of repetitions performed at each RME load was greater (*p* = 0.035) at 20% (0.11 ± 0.09) compared to the 40% (0.08 ± 0.04) condition (*p* = 0.010) (Figure 1c). AME_FC20%_ set CVs were not different (*p* = 0.288) from each other (S1: 0.10 ± 0.06; S2: 0.11 ± 0.06; S3: 0.12 ± 0.06) (Figure 1d). Muscular endurance at each load was different (*p* < 0.001), with a greater number of repetitions performed at each lighter load (20%: 42.7 ± 27.3 repetitions; 30%: 21.0 ± 5.7 repetitions; 40%: 14.4 ± 3.4 repetitions) (*p* < 0.001) (Figure 1a). There was a main effect of set with AME_FC20%_ (*p* = 0.001), where S1 (48.6 ± 33.4 repetitions) was greater than S2 (37.4 ± 18.2 repetitions) (*p* = 0.002) and S3 (36.1 ± 19.0 repetitions) (*p* < 0.001), but S2 and S3 were not different (*p* = 0.557), demonstrating a plateau in muscular endurance (Figure 1b).

### 3.2. Sex and Training Status Effects

**Figure 2:**
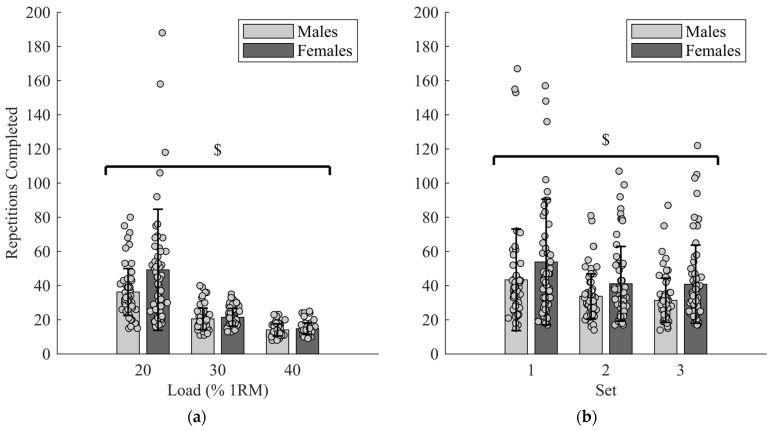
Sex differences in muscle endurance: (**a**) number of repetitions performed by males and females at each of the three external relative loads. $ Significantly more repetitions performed by females; (**b**) number of repetitions performed by males and females at each set of AME_FC20%_. $ Significantly more repetitions performed by females.

Females showed greater muscular endurance on both the variable load (*p* = 0.011) and AME_FC20%_ (*p* = 0.002) legs, with no interaction with load (*p* = 0.090) (Figure 2a) or AME_FC20%_ set (*p* = 0.912) (Figure 2b). Muscular endurance on both the variable load leg (r = 0.139, *p* = 0.006) and AME_FC20%_ leg (r = 0.303, *p* < 0.001) was correlated with MVPA, but there was no correlation between RT and muscular endurance on the variable load (*p* = 0.157) or AME_FC20%_ leg (*p* = 0.053).

## 4. Discussion

Dynamic knee extension muscular endurance showed good-to-excellent repeatability across loads ranging from 20 to 40% of 1RM. Measurement error, expressed in both absolute (SEM and SDD) and relative (ICC and CV) terms, was greater at lower compared with higher loads. Muscular endurance tests performed after warm-ups not involving task failure exhibited lower absolute and relative measurement errors than tests performed 20 min or more following a previous set to failure. The good-to-excellent repeatability of all loads and lack of visit effect suggests that a single familiarization session was sufficient to eliminate any low-load muscular endurance learning effects. In agreement with the previous literature, females and individuals reporting greater MVPA demonstrated higher muscular endurance and better maintenance of performance across repeated sets, while there was no observed effect of RT. To ensure the identification of adaptation in low-load muscular endurance, practitioners should utilize the reported SDD values as a critical threshold to distinguish meaningful individual improvements from inherent variability.

The observed decrease in repeatability with declining loads may reflect greater physiological complexity at loads below ~40% 1RM. The COT for knee extension occurs between 30 and 40% 1RM, with strength and anaerobic capacity explaining most interindividual variation [5]. At lower loads below the COT, there is an increased reliance on mitochondrial oxidative capacity, capillarization per fibre, and slow-twitch fibre composition, in addition to anaerobic performance determinants [12,22]. Furthermore, some authors have suggested a greater importance of mental toughness/discomfort tolerance in determining performance at lower loads [23]. Other studies have shown mixed results, supporting and/or contradicting increases in repeatability with load [2,3,4]. However, these studies all include loads which exceed the COT (~40% 1RM), possibly suggesting that proximity to the COT influences repeatability due to the shift in aerobic/anaerobic performance determinants. Though these mechanisms seem in line with the findings presented here, it is important to note that no physiological measures were taken during this study and therefore this discussion remains speculative.

When examining the repeatability of sets in AME_FC20%_, the inter-set rest period duration has repeatedly been shown to impact performance in subsequent sets [24,25]. Nuzzo [6] suggests that when rest periods are insufficient, 1RM strength is incompletely recovered, causing sequential sets performed at the initial load to become a higher relative fraction of current force production capacity. This persistent fatigue is likely primarily of peripheral origin following resistance exercise below COT [26] and has been shown to persist following a five-minute rest period [27]. In the present study, even a 20 min rest period was insufficient to recover muscular endurance performance to resting levels. Due to limitations of study design, it is not possible to determine the sufficiency of a 20 min rest period following all loads. However, based on both the lower muscular endurance in S2 and S3 in the AME_FC20%_ condition and the reduced repeatability in the variable load leg compared to the AME_FC20%_ leg, it is likely that 20 min is insufficient for full recovery of performance after any set performed to volitional failure below the COT. Previous reports indicate that lower-load contractions performed to task failure induce greater peripheral fatigue than higher-load contractions, suggesting that muscle endurance tests conducted above the COT may require shorter recovery [28,29].

RME estimates at 20, 30, and 40% 1RM differed from the models proposed by Nuzzo et al. [2]. This is likely due to the slow, 2/2 tempo of contraction used in this study and the limited pool of studies investigating these low loads. Although Nuzzo et al. [2] did report whether a metronome was reported in each study, the actual contraction tempo was not included as a moderator. Additionally, most of the studies included did not specify whether a metronome or self-selected tempo was used at all.

The observed sex differences in muscular endurance align with previous findings [30]. Females consistently exhibit greater muscular endurance than males during isometric contractions; however, evidence from dynamic contractions has been less consistent [31,32]. As noted by Nuzzo [33], the magnitude of sex effects depends on factors such as exercise selection, contraction tempo, and load. Indeed, very few studies have examined the effect of sex on muscular endurance during dynamic contractions taken to volitional fatigue. The current body of evidence would suggest that for slow-tempo, single muscle, dynamic contractions such as those performed in this study, females have greater muscular endurance [31,32]. Possible explanations for these sex differences include sexually dimorphic relative fibre type cross-sectional areas, fatigue resistance and contractile mechanisms [31,32,33]. Results from the work presented here suggest that females may have a relative advantage in muscular endurance at lower loads. However, given that the present study was not explicitly designed to examine sex differences, these findings should be interpreted as exploratory and hypothesis generating rather than demonstrative of a sex-based effect.

Aerobic training status also influenced muscular endurance, showing weak correlations with RME and weak-to-moderate correlations with the AME_FC20%_. Consistent with Fliss et al. [5], strength training experience did not appear to affect muscular endurance. This likely reflects the greater reliance on aerobic metabolism during lighter-load tasks, which may be enhanced in individuals with higher aerobic fitness and oxidative muscle phenotypes [34,35,36]. Conversely, resistance training primarily increases maximal strength, which—despite increasing absolute muscular endurance—raises the absolute load used in relative muscular endurance, thus leaving performance unchanged.

## 5. Conclusions

Evaluating the repeatability of commonly used performance measures is essential, particularly when research findings inform practical decision-making. The present investigation examined loads of 20, 30, and 40% 1RM performed in a random order and three sequential sets of 20% 1RM—loads below the COT. It was shown that muscular endurance performance at these loads has good-to-excellent repeatability based on their ICCs, with SEM and SDD also being quantified. Heavier loads and earlier sets were found to be more repeatable than lighter loads or later sets. Researchers and exercise professionals should recognize that even extended rest intervals may not fully eliminate variability when conducting multiple exhaustive exercise tests within the same day. Additionally, more prolonged lighter-load tasks performed to failure likely require longer recovery than shorter high-load tasks. The present study demonstrates that dynamic knee extension muscular endurance can be reliably assessed when testing sessions are separated by at least 48 h following a single familiarization visit. Therefore, for the most consistent results, practitioners should ensure participants are familiarized with each specific test and schedule assessments on separate days. Furthermore, if repeatability is a priority, practitioners are encouraged to use heavier resistance loads for individual assessments and perform the highest priority tests first. To ensure clinical significance, practitioners should only recognize an individual’s muscular endurance gains when they exceed the SDD; any increase below this threshold may simply reflect measurement error. Reporting and interpreting SDDs is therefore encouraged, as these values allow practitioners to distinguish true performance changes from normal measurement variability.

## Figures and Tables

**Table 1:** Participant descriptive statistics and visit spacing.

Characteristic	Overall, n = 40 ^1^	Males, n = 20 ^1^	Females, n = 20 ^1^
Age (yrs)	21.2 ± 2.1	21.2 ± 1.9	21.2 ± 2.2
Height (cm)	171.2 ± 9.8	177.9 ± 7.7	164.7 ± 6.8 *
Weight (kg)	71.4 ± 12.5	79.1 ± 11.3	63.8 ± 8.3 *
Variable Load Leg 1RM (kg)	44.3 ± 11.2	51.4 ± 10.3	37.2 ± 6.8 *
AME_FC20%_ Leg 1RM (kg)	44.3 ± 11.4	51.9 ± 10.2	36.6 ± 6.5 *
MVPA (min/wk)	121.4 ± 125.8	106.8 ± 120.1	136.1 ± 129.6
LIPA (min/wk)	199.3 ± 158.0	202.3 ± 190.6	196.3 ± 116.6
RT (min/wk)	166.1 ± 167.6	154.1 ± 175.5	178.1 ± 158.4
Visit Spacing (days)	5.7 ± 3.2	5.0 ± 3.0	6.3 ± 3.3
ΔTime of Testing (hours)	1.9 ± 1.7	2.1 ± 1.8	1.8 ± 1.5

^1^ Values shown as mean ± SD. * Significantly different from males (*p* < 0.001).

**Table 2:** ICCs and absolute repeatability of all loads.

Exercise Condition	Load/Set	ICC (95% CI) ^1^	SEM	SDD
RME Loads	20% 1RM	0.77 (0.64–0.86)	2.40	6.66
	30% 1RM	0.78 (0.67–0.87)	0.93	2.59
	40% 1RM	0.82 (0.72–0.89)	0.47	1.29
AME_FC20%_ Sets	Set 1	0.94 (0.90–0.97)	1.28	3.54
	Set 2	0.88 (0.82–0.93)	1.42	3.94
	Set 3	0.86 (0.77–0.92)	1.74	4.82

^1^ ICC repeatability: <0.5 = poor, 0.5–0.75 = moderate, 0.75–0.9 = good, >0.9 = excellent [10]. Relative muscle endurance (RME); intraclass correlation coefficient (ICC); confidence interval (CI); standard error of the measurement (SEM); smallest detectable difference (SDD).

## Data Availability

The raw data supporting the conclusions of this article will be made available by the authors on request.

## Abbreviations

The following abbreviations are used in this manuscript:

1RM	1 repetition maximum
SEM	Standard error of the measurement
SDD	Smallest detectable difference
ICC	Intraclass correlation coefficient
CI	Confidence interval
RME	Relative muscle endurance
AME_FC20%_	20% 1RM fatigue curve
CV	Coefficient of variation
20%	RME 20% 1RM set
30%	RME 30% 1RM set
40%	RME 40% 1RM set
S1	First AME_FC20%_ set
S2	Second AME_FC20%_ set
S3	Third AME_FC20%_ set
MVPA	Moderate-to-vigorous physical activity minutes per week
RT	Resistance training minutes per week
LIPA	Low-intensity physical activity minutes per week
COT	Critical occluding tension

## References

[1] Schoenfeld B.J., Grgic J., Van Every D.W., Plotkin D.L. (2021). Loading recommendations for muscle strength, hypertrophy, and local endurance: A re-examination of the repetition continuum. Sports.

[2] Nuzzo J.L. (2024). Maximal number of repetitions at percentages of the one repetition maximum: A meta-regression and moderator analysis of sex, age, training status, and exercise. Sports Med..

[3] Hoeger W.W.K., Hopkins D.R., Barette S.L., Hale D.F. (1990). Relationship between repetitions and selected percentages of one repetition maximum: A comparison between untrained and trained males and females. J. Strength Cond. Res..

[4] Mitter B., Csapo R., Bauer P., Tschan H. (2022). Reproducibility of strength performance and strength-endurance profiles: A test-retest study. PLoS ONE.

[5] Fliss M.D., Abercrombie M.J., Denson K.G., Wiens L., Losciale J.M., Schweitzer A.M., Coccimiglio I.F., Tripp T.R., Burr J.F., MacInnis M.J. (2025). A critical occluding tension phase transition occurs between 30% and 40% 1RM in dynamic knee extension exercise. Scand. J. Med. Sci. Sports.

[6] Nuzzo J.L. (2024). Muscle strength preservation during repeated sets of fatiguing resistance exercise: A secondary analysis. J. Strength Cond. Res..

[7] Hopkins W.G. (2000). Measures of reliability in sports medicine and science. Sports Med..

[8] Swinton P.A., Hemingway B.S., Saunders B., Gualano B., Dolan E. (2018). A statistical framework to interpret individual response to intervention: Paving the way for personalized nutrition and exercise prescription. Front. Nutr..

[9] Beckerman H., Roebroeck M.E., Lankhorst G.J., Becher J.G., Bezemer P.D., Verbeek A.L.M. (2001). Smallest real difference, a link between reproducibility and responsiveness. Qual. Life Res..

[10] Koo T.K., Li M.Y. (2016). A guideline of selecting and reporting intraclass correlation coefficients for reliability research. J. Chiropr. Med..

[11] Ward S.R., Davis J., Kaufman K.R., Lieber R.L. (2007). Relationship between muscle stress and intramuscular pressure during dynamic muscle contractions. Muscle Nerve.

[12] El Bojairami I., Driscoll M. (2022). Correlating skeletal muscle output force and intramuscular pressure via a three-dimensional finite element muscle model. J. Biomech. Eng..

[13] Azuma K., Homma S., Kagaya A. (2000). Oxygen supply-consumption balance in the thigh muscles during exhausting knee-extension exercise. J. Biomed. Opt..

[14] Hultman E., Sjöholm H. (1983). Energy metabolism and contraction force of human skeletal muscle in situ during electrical stimulation. J. Physiol..

[15] Greenhaff P.L., Söderlund K., Ren J.M., Hultman E. (1993). Energy metabolism in single human muscle fibres during intermittent contraction with occluded circulation. J. Physiol..

[16] Cunniffe B., Sharma V., Cardinale M., Yellon D. (2017). Characterization of muscle oxygenation response to vascular occlusion: Implications for remote ischaemic preconditioning and physical Performance. Clin. Physiol. Funct. Imaging.

[17] Wernbom M., Augustsson J., Thomeé R. (2006). Effects of vascular occlusion on muscular endurance in dynamic knee extension exercise at different submaximal loads. J. Strength Cond. Res..

[18] Wernbom M., Järrebring R., Andreasson M.A., Augustsson J. (2009). Acute effects of blood flow restriction on muscle activity and endurance during fatiguing dynamic knee extensions at low load. J. Strength Cond. Res..

[19] Grgic J., Lazinica B., Schoenfeld B.J., Pedisic Z. (2020). Test–retest reliability of the one-repetition maximum (1RM) strength assessment: A systematic review. Sports Med. Open.

[20] Warburton D.E.R., Jamnik V.K., Bredin S.S.D., Gledhill N. (2011). The physical activity readiness questionnaire for everyone (PAR-Q+) and electronic physical activity readiness medical examination (ePARmed-X+). Health Fit. J. Can..

[21] Lazic S.E. (2008). Why we should use simpler models if the data allow this: Relevance for ANOVA designs in experimental biology. BMC Physiol..

[22] van der Zwaard S., van der Laarse W.J., Weide G., Bloemers F.W., Hofmijster M.J., Levels K., Noordhof D.A., de Koning J.J., de Ruiter C.J., Jaspers R.T. (2018). Critical determinants of combined sprint and endurance performance: An integrative analysis from muscle fiber to the human body. FASEB J..

[23] Fisher J., Steele J., Smith D. (2017). High- and low-load resistance training: Interpretation and practical application of current research findings. Sports Med..

[24] Millender D.J., Mang Z.A., Beam J.R., Realzola R.A., Kravitz L. (2021). The effect of rest interval length on upper and lower body exercises in resistance-trained females. Int. J. Exerc. Sci..

[25] Senna G.W., Rodrigues B.M., Sandy D., Scudese E., Bianco A., Dantas E.H.M. (2017). Heavy vs. light load single-joint exercise performance with different rest intervals. J. Hum. Kinet..

[26] Walker S., Davis L., Avela J., Häkkinen K. (2012). Neuromuscular fatigue during dynamic maximal strength and hypertrophic resistance loadings. J. Electromyogr. Kinesiol..

[27] Hureau T.J., Broxterman R.M., Weavil J.C., Lewis M.T., Layec G., Amann M. (2022). On the role of skeletal muscle acidosis and inorganic phosphates as determinants of central and peripheral fatigue: A 31P-MRS study. J. Physiol..

[28] Behm D.G., St-Pierre D.M. (1997). Effects of fatigue duration and muscle type on voluntary and evoked contractile properties. J. Appl. Physiol..

[29] Marshall P.W., Forward T., Enoka R.M. (2022). Fatigability of the knee extensors following high- and low-load resistance exercise sessions in trained men. Eur. J. Appl. Physiol..

[30] Maughan R.J., Harmon M., Leiper J.B., Sale D., Delman A. (1986). Endurance capacity of untrained males and females in isometric and dynamic muscular contractions. Eur. J. Appl. Physiol. Occup. Physiol..

[31] Hunter S.K. (2016). Sex differences in fatigability of dynamic contractions. Exp. Physiol..

[32] Hunter S.K. (2016). The relevance of sex differences in performance fatigability. Med. Sci. Sports Exerc..

[33] Nuzzo J.L. (2023). Narrative review of sex differences in muscle strength, endurance, activation, size, fiber type, and strength training participation rates, preferences, motivations, injuries, and neuromuscular adaptations. J. Strength Cond. Res..

[34] Campos G.E., Luecke T.J., Wendeln H.K., Toma K., Hagerman F.C., Murray T.F., Ragg K.E., Ratamess N.A., Kraemer W.J., Staron R.S. (2002). Muscular adaptations in response to three different resistance-training regimens: Specificity of repetition maximum training zones. Eur. J. Appl. Physiol..

[35] MacInnis M.J., Gibala M.J. (2017). Physiological adaptations to interval training and the role of exercise intensity. J. Physiol..

[36] Reilly T., Morris T., Whyte G. (2009). The specificity of training prescription and physiological assessment: A review. J. Sports Sci..

